# The Influence of Basil and Cinnamon Essential Oils on Bioactive Sponge Composites of Collagen Reinforced with Hydroxyapatite

**DOI:** 10.3390/ma18030626

**Published:** 2025-01-30

**Authors:** Alina Robu, Madalina Georgiana Albu Kaya, Aurora Antoniac, Durmuș Alpaslan Kaya, Alina Elena Coman, Maria-Minodora Marin, Robert Ciocoiu, Rodica Roxana Constantinescu, Iulian Antoniac

**Affiliations:** 1 Faculty of Materials Science and Engineering, National University of Science and Technology Politehnica, 060042 Bucharest, Romania; alinarobu2021@gmail.com (A.R.); ciocoiurobert@gmail.com (R.C.); antoniac.iulian@gmail.com (I.A.); 2 National Research and Development Institute for Textiles and Leather, Collagen Department, 93 Ion Minulescu, 031215 Bucharest, Romania; coman.alina27@yahoo.com; 3 Department of Field Crops, Faculty of Agriculture, Hatay Mustafa Kemal University, Antakya-Hatay 31034, Turkey; dak1976@msn.com; 4 Advanced Polymer Materials Group, National University of Science and Technology Politehnica, 060042 Bucharest, Romania; minodora.marin@ymail.com; 5 INCDTP—Leather & Footwear Research Institute, Biotechnologies and Environment Protection Research Department, 031215 Bucharest, Romania; rodica.roxana@yahoo.com

**Keywords:** wound dressing, collagen lyophilization, basil essential oil, cinnamon essential oil, antibacterial activities

## Abstract

The increasing prevalence of acute traumas, surgical wounds, and chronic skin wounds poses significant therapeutic challenges for wound treatment. One of the main concerns in wound care is the danger of infection, which is a significant barrier to healing and a cause of higher morbidity and mortality rates. The emergence of drug-resistant bacterial species is becoming more frequent every day. Antimicrobial dressings have become a viable strategy for wound healing and hospital expense savings. Several factors, such as the wound’s localization and state, microbial load, and cost, must be considered when choosing an appropriate antimicrobial dressing. One of the key goals of wound care is infection avoidance. This study addresses the therapeutic challenges of acute traumas, surgical wounds, and chronic skin wounds, focusing on infection prevention and combating drug-resistant bacterial strains. The research explores the development of novel composite wound dressings incorporating hydroxyapatite, known for its osteoconductive properties, and essential oils from basil and cinnamon, recognized for their antimicrobial activity. The study evaluates the impact of these additives on key properties such as surface morphology, water absorption, enzymatic degradation, and mechanical performance. Antimicrobial tests showed that two experimental samples (A1S and A1BS) exhibited significant activity against *Escherichia coli* but not on *Staphylococcus aureus*. The results highlight the dressings’ enhanced antimicrobial properties, mechanical strength, and controlled degradation, making them promising candidates for advanced wound healing. Tailored applications were identified, with each dressing composition offering unique benefits for specific wound-healing scenarios based on the balance between flexibility, structural support, and bioactivity.

## 1. Introduction

Bioactive wound dressings are increasingly used in wound healing and tissue engineering because they support tissue regeneration and provide antimicrobial protection. Antimicrobial dressings are used for acute, non-healing wounds that are infected or in danger of infection. The antimicrobial agent used, the wound’s properties, and how it is delivered all influence the dressing selection. Certain antimicrobial dressings solely have an antiseptic agent; they do not include any antibiotics. Although antiseptic solutions are excellent at killing microorganisms, they can also harm or destroy healthy tissue. As a result, its application in wound care has been restricted to lessening the impact of infections on the skin. The antibacterial properties of wound dressings are usually derived from essential oils, silver, copper, iodine, or polyhexamethylene biguanide [1,2,3,4,5]. Type I collagen is the current gold standard in tissue engineering [6] due to its biocompatibility, biodegradability, bioresorbability, and hemostatic activity. Because of these properties, various medical applications use collagen as a carrier of bioactive components in various forms, such as sponges, gels, hydrogels, powders, solutions, films, and matrices. However, collagen-based materials often require additional components to enhance their functional properties [7]. Antimicrobial collagen-based biomaterials are appealing for tissue engineering, implanted device components, wound dressings, and other applications [6].

Wound dressings with antibacterial effects are used to prevent infections and promote healing by incorporating various antimicrobial agents and biomaterials [8,9,10,11]. Common agents include silver nanoparticles, manuka honey, chitosan, iodine, essential oils (tea tree, eucalyptus, thyme, and lavender), metal oxides (zinc oxide, titanium dioxide), and plant extracts (Aloe vera, neem, curcumin) [12,13,14,15,16,17,18,19,20]. Synthetic agents like polyhexamethylene biguanide (PHMB) are also used for broad-spectrum activity. Biomaterials such as hydrogels (polyvinyl alcohol (PVA), polyethylene glycol (PEG), chitosan), hydrocolloids (carboxymethyl cellulose (CMC), gelatin, pectin), nanofibers (polycaprolactone (PCL), poly (lactic-co-glycolic acid) (PLGA)), alginates (calcium and sodium alginate), polyurethane foam, and collagen are utilized for their absorbent and antimicrobial properties [21,22,23,24,25]. Due to the rise of multidrug-resistant (MDR) bacteria caused by the overuse of antibiotics, research has shifted toward non-antibiotic agents like silver and essential oils, which have broad-spectrum activity and are effective against biofilms [1,5,14,15,16,17,20,26,27,28,29,30,31,32]. Essential oils are particularly favored for their ability to disrupt bacterial cell membranes and inhibit resistance development, making them sustainable alternatives with added anti-inflammatory and healing benefits [33,34,35,36].

**Figure 1:**
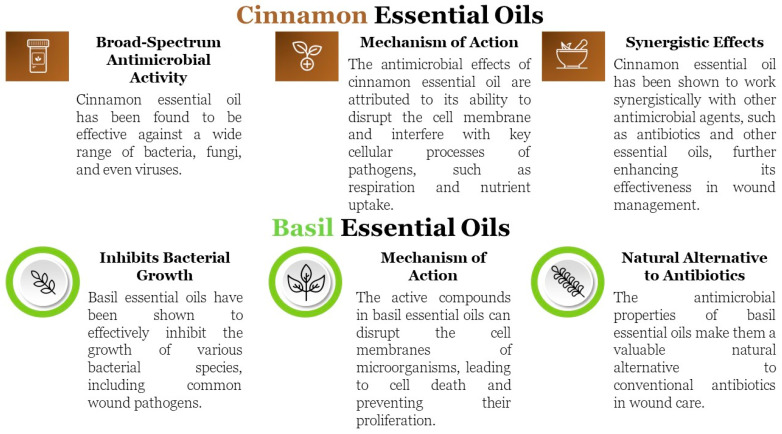
Antimicrobial Effects of Basil and Cinnamon Essential Oils.

Essential oils (EOs) are natural oils obtained primarily through distillation, containing volatile compounds with antimicrobial, antifungal, and antioxidant properties. They are extracted from various plant parts (flowers, seeds, leaves, etc.), yielding different properties. Used for thousands of years in perfumes, food flavoring, and treating illnesses, EOs are easily absorbed by the body due to their fat solubility [37]. Basil essential oil, extracted from the leaves and blossoms of *Ocimum basilicum*, has important properties (Figure 1). It is known for its antimicrobial, anti-inflammatory, and antioxidant properties, making it valuable in wound care [38,39,40,41,42]. Key components like linalool, methyl chavicol, and eugenol contribute to its therapeutic effects, including inhibiting the growth of bacteria such as *E. coli* and *Staphylococcus* species by disrupting bacterial cell membranes [40,41,42]. Basil essential oil also reduces inflammation by blocking inflammatory mediators, aiding in wound healing by promoting the proliferation of skin cells, collagen deposition, and angiogenesis [39,43,44]. Basil essential oils help hasten the healing process and encourage the appropriate course of wound repair by reducing the inflammatory reaction [45]. The effectiveness of basil essential oils as adjuvant therapy for wound care, particularly for chronic or difficult-to-heal wounds, has been demonstrated by several clinical studies [15,16,45,46]. Basil essential oils can be used with other therapeutic agents or conventional wound care techniques to optimize their ability to heal [47,48,49]. Basil essential oils can also provide a thorough and all-encompassing approach to wound management when combined with compression therapy, debridement, and other common wound care methods. Studies have demonstrated that *Ocimum basilicum* essential oil exhibits significant antibacterial activity against both Gram-positive and Gram-negative bacteria. Notably, it has shown greater efficacy than commercial antibiotics such as ciprofloxacin and gentamicin against certain strains, including coagulase-positive staphylococci [50].

Cinnamon essential oil is derived from the aromatic bark of the cinnamon tree, a spice prized for its distinct flavor and fragrance. This versatile oil has a long history of medicinal use, particularly in treating wounds and skin ailments. The oil’s strong antibacterial, anti-inflammatory, and wound-healing qualities are attributed to ingredients such as linalool, eugenol, and cinnamaldehyde [51,52]. Cinnamon EO also has been reported to possess potent antibacterial properties [53,54,55]. This antibacterial activity is essential in preventing infection and keeping the wound area clean.

Combining basil essential EO and cinnamon in bioactive collagen dressings has considerable potential to accelerate wound healing. The antimicrobial, anti-inflammatory, and antioxidant properties of these essential oils can help reduce the risk of infection, reduce local inflammation, and stimulate tissue regeneration.

When compared to other plant-derived essential oils, both basil and cinnamon oils demonstrate robust antibacterial activities against multidrug-resistant bacteria, including *Staphylococcus aureus,* often surpassing the efficacy of certain commercial antibiotics and other plant-derived essential oils [53,56,57,58]. Their potential synergistic effects when combined with conventional antibiotics further highlight their promise as complementary agents in antimicrobial therapies. In conclusion, essential oils of basil and cinnamon are two key ingredients that can improve the effectiveness of bioactive collagen dressings. Recent studies have shown that these essential oils have the potential to accelerate wound healing through their antimicrobial, anti-inflammatory, and antioxidant action. The use of these bioactive dressings may represent an innovative and effective approach to the treatment of chronic wounds and skin lesions.

To incorporate essential oils (EOs) into bioactive wound dressings, hydroxyapatite (HAp) is used as a critical component because it provides structural compatibility with both collagen and the volatile nature of EOs [59]. Hydroxyapatite, used in small concentrations (e.g., 1–5%), helps incorporate essential oils into a stable, bioactive matrix suitable for wound dressings. The combination of collagen with HAp provides a scaffold that retains the EOs’ bioactivity, allowing for sustained release at the wound site [60]. Collagen and hydroxyapatite combinations have been widely researched due to their natural compatibility with human tissues [61,62]. HAp is used at much lower concentrations for wound dressings compared to bone applications to avoid excessive mineral content that might hinder the flexibility required for wound healing. Collagen-HAp foam dressings have been used for their absorbent properties and the ability to maintain a moist environment that supports faster healing. These dressings often incorporate antimicrobial agents such as essential oils. Lyophilization of collagen in the manufacturing process of bioactive dressings is essential to preserve the integrity and effectiveness of collagen in treating wounds, providing a dressing that aids in faster healing and protects against infection. By lyophilization, collagen is dried in a way that preserves its essential properties to function effectively in bioactive dressings. This gentle drying process prevents collagen from being damaged, maintaining its ability to bind and moist wounds, promote healing, and protect against infection.

Antimicrobial resistance occurs when microorganisms develop the ability to survive exposure to antimicrobial agents that would normally inhibit their growth or kill them. The basil and cinnamon essential oils proposed for use in this research are known to contain bioactive compounds such as eugenol, cinnamaldehyde, and linalool that disrupt resistance mechanisms, and they interact with the lipid bilayer of microbial cell membranes, increasing permeability and causing leakage of cellular contents. By targeting the structural and biochemical vulnerabilities of bacteria, basil and cinnamon EOs could mitigate the risk of resistance development, making the dressing effective against a broad spectrum of pathogens. Adhesion of a dressing to the skin is essential to ensure adequate wound coverage, prevent contamination, and minimize dressing displacement. Collagen-based dressings are naturally adhesive due to their biocompatibility and ability to conform to the wound surface. Hydroxyapatite, incorporated into the collagen matrix, increases the surface roughness, improving mechanical interconnection with the skin and wound site [63,64,65]. This promotes better adhesion while maintaining ease of removal to avoid secondary trauma. Minimal reports of irritation in previous EO-based products suggest good tolerability [66].

The inclusion of basil and cinnamon EOs in hydroxyapatite-reinforced collagen matrices supports wound healing through synergistic biological activities: collagen provides a biomimetic environment that supports fibroblast adhesion, proliferation, and migration, critical for tissue repair, HAp has the role of both preserving the properties of the essential oils and supporting the structural integrity of the wound site, acting as a reinforcement for soft tissue, and can provide mechanical support without compromising flexibility. Bioactive compounds in basil and cinnamon EOs, such as eugenol and cinnamaldehyde, exhibit anti-inflammatory properties supporting the transition to the healing phase. EOs reduce oxidative stress at the wound site by protecting cellular components from oxidative damage and enhancing cell survival. Basil and cinnamon EOs do not interfere with the mechanical properties of the dressing, as demonstrated in studies of polymeric materials incorporating essential oils [67]. The antimicrobial properties of EOs reduce microbial load, preventing infections that can delay healing and promoting an environment conducive to tissue regeneration.

Since a smoother surface displays biofouling features, it is well-recognized that a big surface with high roughness is advantageous to microbial attachment [68].

Following the testing of five essential oils (cinnamon, myrtle, frankincense, chamomile, and basil) within our research group, it emerged that cinnamon and basil essential oils have the most promising potential to be used for bioactive wound dressings with hydroxyapatite-reinforced collagen matrix.

Taking into account all the information presented previously, the following research was started with the aim of developing a composite bioactive wound dressing incorporating HAp and EOs from basil and cinnamon to explore their potential synergistic effects.

## 2. Materials and Methods

### 2.1. Preparation of Biopolymeric Samples

The following medical-grade ingredients were used to generate the collagen-based composite compositions to be used for skin lesion healing: collagen gel with an initial concentration of 1.77% (*w*/*w*) and with a pH of 1.8 was prepared from calfskin in conformity with the actual method established and used at the Collagen Research Department of the Division of Leather and Footwear Research Institute [69], hydroxyapatite (HAp) with purity p.a., ≥90% from (Sigma-Aldrich Chemie GmbH, Taufkirchen Germany), essential oils (EOs) (cinnamon EO obtained from *Cinnamomum verum* bark, and basil EO obtained from *Ocimum basilicum* leaf). Distilled water served as the solvent for preparing the experimental samples. Because the essential oils are volatile, we mixed the same amount of HAp with different quantities of essential oils: 1% hydroxyapatite and 1% essential oil (except reference sample, R, which consists of 1% collagen and 1% HAp) were added to the collagen gel (with an initial concentration of 1.77%). The composite gels were adjusted to 7.4 pH with sodium hydroxide 1 M and 15 mL of water (under stirring). The sample with 1% basil essential oil was marked as A1B, that with 1% cinnamon essential oil was marked A1C, and the one with both essential oils was named A1BC as is presented in Table 1. These composite gels were cross-linked with glutaraldehyde (0.05%) and finally put into glass Petri dishes with a diameter of 5 cm and a height of 1 cm. After that, they were placed in a lyophilizer, previously cooled to −40 °C, for 90 min.

**Figure 2:**
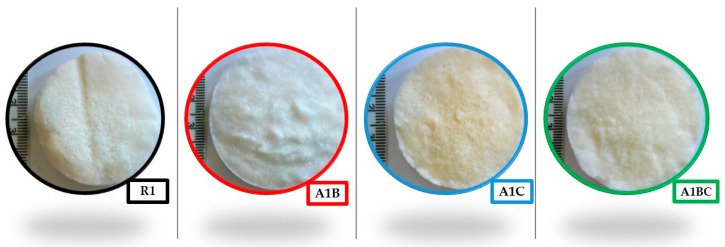
Macroscopic images of the R1, A1C, A1B, and A1BC sponges.

The lyophilization process lasted 48 h [69], obtaining samples corresponding to the prepared composite gels (Figure 2), which were subsequently characterized.

### 2.2. Fourier-Transform Infrared Spectroscopy (FTIR)

Fourier-transform infrared spectroscopy (FTIR) measurements on prepared samples were carried out using an attenuated total reflectance (ATR) accessory on a Vertex 70 Bruker FTIR spectrometer (Billerica, MA, USA). The FTIR spectra in the 400–4000 cm^−1^ wavenumber region were registered at 4 cm^−1^ resolution for every manufactured formulation using the ATR-FTIR mode.

### 2.3. Scanning Electron Microscopy (SEM)

The microstructures of the composite samples with a polymeric matrix, hydroxyapatite, and essential oil were characterized using a Quattro S scanning electron microscope (Thermo Fisher Scientific, Hillsboro, OR, USA).

### 2.4. Water Absorption

Samples measuring 1 cm × 1 cm × 0.8 cm were first weighed to determine the initial mass Wt. They were then immersed in 3 mL of distilled water at 37 °C and subsequently weighed at different time intervals (30 min and 1, 2, 3, 24, 48, and 72 h).

As documented in the literature [70,71], the measurements of the swelling process obtained at each previously mentioned time interval were used to calculate the water absorption capacity using Equation (1).

(1) $$W_{a}=\frac{W_{t}-W_{d}}{W_{t}}\;[g/g]$$

where: *W_d_* is the initial mass, *W_t_* is the mass of the immersed sample at time *t*. For increased accuracy, the analysis was performed in triplicate.

### 2.5. Enzymatic Degradation

The stages of the degradation mechanism initially consist of a hydration process followed by loss of strength properties, loss of integrity, and, finally, a loss of mass process. A degradation in collagenase was performed in vitro to simulate the behavior of the materials under in vivo conditions and to observe the biological stability. Enzymatic degradation was assessed by immersing pieces of specimens (already swelled in water) in *Clostridium hystoliticum* collagenase solution (10^−6^ mg/mL prepared in PBS at 37 °C) and monitoring the degradation over time (4, 8, 24, 28, 32, 34 h). The measurements were performed in triplicate. The samples were maintained at 37 °C for up to 34 h. The following formula was used:

(2) $$Weight\;loss\;\left[\%\right]=\frac{W_{i}-W_{t}}{W\dot{t}}\cdot 100$$

where: *Wi* represents the initial mass and *Wt* represents the mass of the sample at time *t*.

### 2.6. Compression Test

The compression test used an Instron 34TM-10 testing machine from Norwood, MA, USA and ASTM D695 [72] as a guide. The test speed was 1 mm/min, and the test’s stopping criterion reached a displacement of 10 mm. No preload was applied before the test; the curves were compensated by eliminating the nose—the area where the material “sits”. The elastic modulus of the samples was determined by linear regression, and the resistance criterion was evaluated by the yield limit determined analogously to the conventional yield limit for a deformation of 0.2%. The results are presented as the average value ± one standard deviation and the statistical tests performed were the one-way ANOVA test followed by Tukey and Fisher pairwise comparisons with a significance level α = 0.05.

### 2.7. Shore C Hardness

Shore C hardness was determined using a DML DH105C Shore C durometer (Sheffield, South Yorkshire, UK) according to ASTM D-2240 specifications [73]. To determine the Shore C hardness of the skin, the determinations were made on an adult’s forearm.

### 2.8. Roughness

The device used to determine surface roughness is a Form Talysurf^®^ i-Series PRO Range from Taylor Hobson (Leicester, UK). The roughness meter comprises a transducer with a standard probe for measuring flat surfaces and uses Metrology 4.0 software (Metrology 4.0 software). Studying the roughness of a biomaterial’s surface can give information about its behavior in the human body. Surface roughness was evaluated using parameters such as Ra (roughness average) and Rt (the distance between the maximum and minimum point of the profile within the evaluation length).

### 2.9. Antimicrobial Activity

Three samples, each with the same mass (0.08 g), were aseptically cut from each biomaterial inside a microbiological hood with laminar flow.

A suspension containing 1.5 × 10^8^ colony-forming units (CFU) was prepared from fresh cultures of *Escherichia coli* (ATCC 10536) and *Staphylococcus aureus* (ATCC 6538 ) under sterile conditions. A volume of 200 μL from each suspension was used to inoculate 90 mm Petri dishes with Mueller–Hinton agarized culture media. In each Petri dish inoculated with the tested microorganism, a sample of each biomaterial was aseptically placed at the center. All Petri dishes prepared in this manner were incubated at 37 °C for 18–24 h. The antimicrobial effect was quantified by measuring the inhibition diameter observed in each case, subtracting the equivalent diameter of the tested biomaterial. Local antimicrobial activity was defined as an average inhibition diameter of less than 5 mm. Moderate activity was attributed to an average inhibition diameter between 6 and 10 mm, while strong activity was assigned to an average inhibition diameter greater than 11 mm. For the comparison of antimicrobial activity, standardized antibiotic discs of ampicillin (AMP 2.5), tobramycin (TOB 10), ticarcillin–clavulanate (TCC 75/10), and gentamicin (GEN 10) (Bio-Rad Lab., Hercules, CA, USA) were used. All tests were performed in triplicate. The results are presented as the average inhibition diameter (Φ) in mm, with its corresponding standard deviation.

## 3. Results and Discussion

### 3.1. Fourier-Transform Infrared Spectroscopy (FTIR)

**Figure 3:**
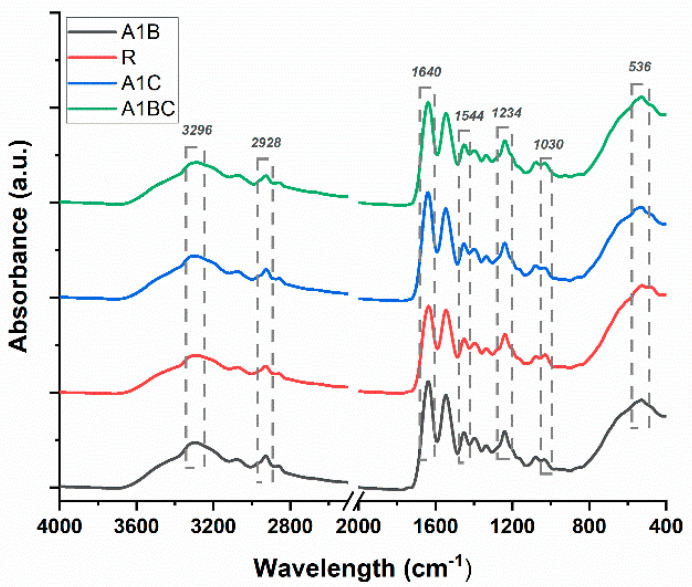
FTIR spectra for R1, A1C, A1B, and A1BC composite sponges.

The FTIR-ATR technique evaluated the chemical structure and interactions between collagen, hydroxyapatite, basil essential oil, and cinnamon essential oil in the obtained composite samples (Figure 3).

For all the analyzed samples, characteristic bands for collagen and hydroxyapatite can be observed, but no changes by the introduction of essential oils in their composition can be noticed.

In the case of collagen, characteristic bands due to stretching vibrations of the N-H bonds in amide A, C-H bonds in amide B, and C=O bonds in amide I are visible at 3296 cm^−1^, 2928 cm^−1^, and 1640 cm^−1^, respectively. At 1544 cm^−1^ and 1234 cm^−1^, bands appear due to deformation vibrations of the N–H bonds in amide II and N–H bonds in amide III [62,74,75,76]. Distinctive bands at 1030 cm^−1^ and 536 cm^−1^ associated with symmetric stretching vibrations of the ${PO}_{4}^{3-}$ group from hydroxyapatite can also be observed [74,75,76,77]. The FTIR spectra of the collagen/HAp/essential oil samples showed that the composite material was successfully manufactured.

### 3.2. Scanning Electron Microscopy (SEM)

**Figure 4:**
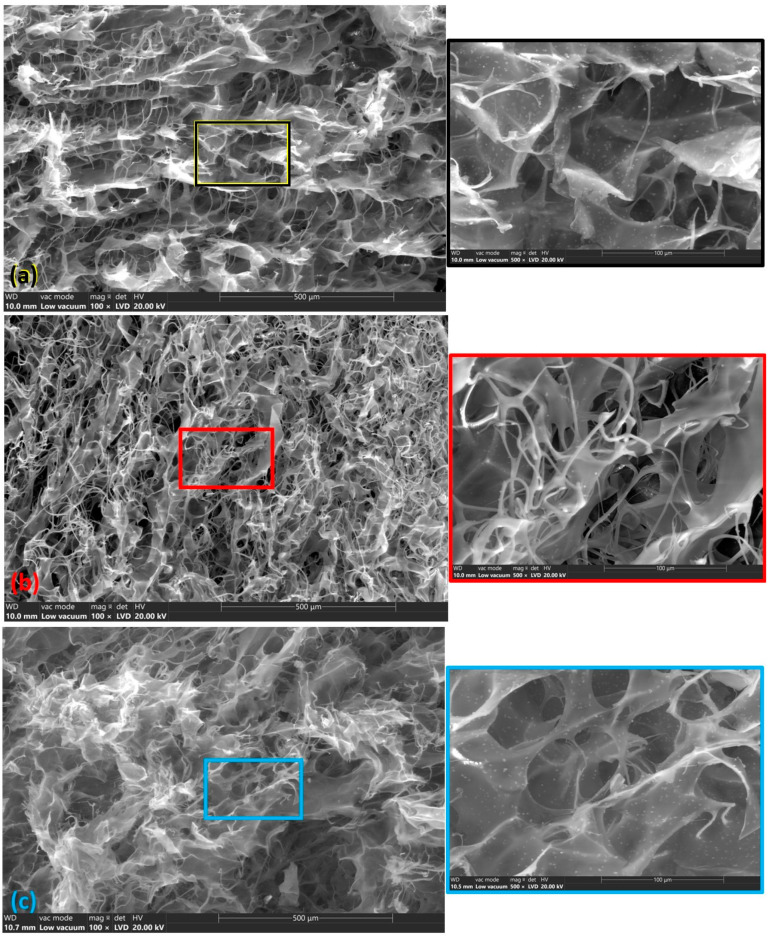

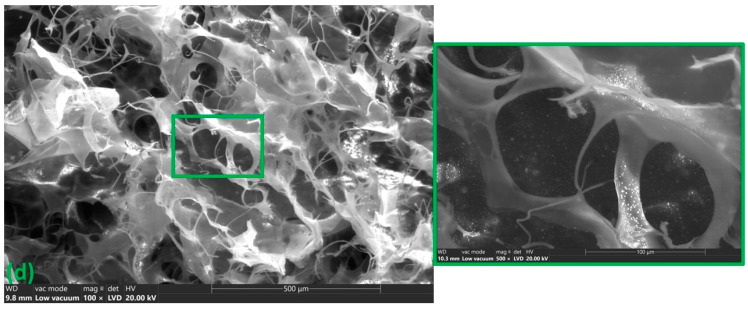
The structure of the composite sponges (at 2 magnifications 100× and 500×): (**a**) R1, (**b**) A1B, (**c**) A1C, and (**d**) A1BC.

SEM analysis is a powerful tool to investigate the morphology and microstructure of materials. In the case of experimental wound dressings, the SEM images will provide detailed information about the surface texture, porosity, component distribution (collagen, hydroxyapatite, basil EO, and cinnamon EO), pore sizes, and how these properties might influence the dressing’s functionality. The reference sample (R1—Figure 4a), composed of collagen and hydroxyapatite, shows a porous, fibrous structure characteristic of collagen with hydroxyapatite particles dispersed within the matrix (detailed image) [78,79]. The tape-shaped collagen fibers appear as elongated, interwoven strands, providing a scaffold-like structure. Porosity is important for fluid absorption and promoting cellular infiltration, which supports tissue healing [80]. The hydroxyapatite particles used are small in size and have a uniform distribution in the collagen matrix. The R1 sample has a porous structure with interconnected micro- and macropores and a smooth surface, which began to be modified with the incorporation of HAp [60]. The addition of basil EO in sample A1B (Figure 4b) influences the sample structure by highlighting a phase separation where the basil EO and collagen–hydroxyapatite matrix form distinct domains, visible as different textures [81]. In the detailed image in Figure 4a, the collagen has a band-like appearance and, in this experimental sample (Figure 4b), a separation of the collagen fibers and a relatively uniform distribution of the bands can be observed.

A relatively uniform distribution of hydroxyapatite particles was also observed. Like basil EO, adding cinnamon EO could modify the surface by forming a hydrophobic layer or creating regions with a smoother texture due to the oil’s presence. Cinnamon oil, composed of hydrophobic compounds like cinnamaldehyde, shows a smoother region in the SEM images. The SEM images reveal a more compact structure with slightly reduced porosity than the R1 sample. The sample A1BC exhibits the most significant changes in surface morphology due to the combination of basil and cinnamon essential oils. The SEM images show a more homogeneous hydrophobic layer, where the essential oils coat the collagen–hydroxyapatite matrix, resulting in a smooth, light, non-porous surface. The SEM reveals minimal porosity, as the oils may block or fill most available pores, making the surface even more compact and smoother than that of the individual basil or cinnamon EO samples.

### 3.3. Water Absorption

**Figure 5:**
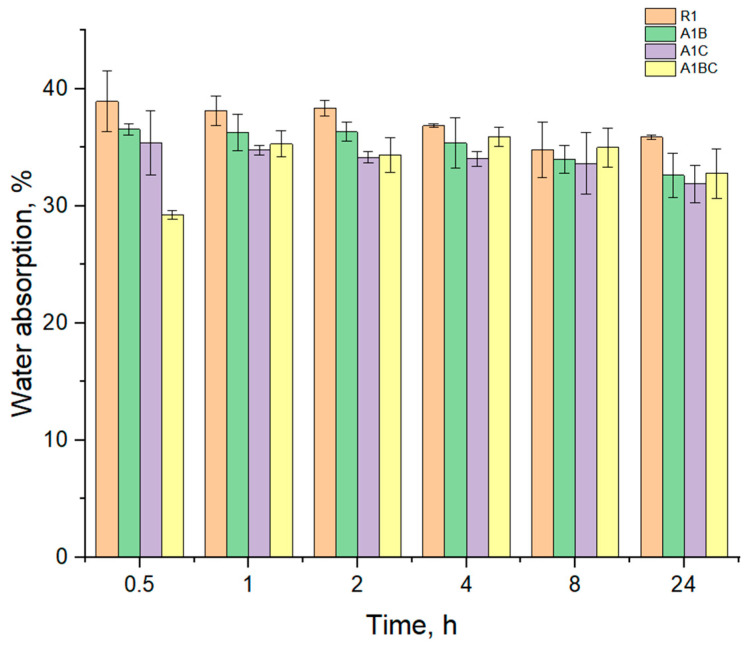
Water absorption values for the investigated samples over time.

The degree of hydration (water absorption capacity) is essential for bioactive dressings to ensure optimal wound healing and maintain patient comfort. Figure 5 shows graphically the variation of the mass of water absorbed as a function of time, evaluated in distilled water at a temperature of 37 °C for 24 h, for all experimental samples obtained from collagen with hydroxyapatite and essential oils. It is observed that the highest amount of water is absorbed in the first 30 min for all the samples, about 29 g/g to 38 g/g.

The degree of hydration, or water absorption capacity, plays a critical role in the performance of wound dressings, particularly in their ability to maintain a moist environment, which is essential for effective wound healing. Since R1 contains only collagen and hydroxyapatite, its water absorption capacity is relatively stable, as collagen and hydroxyapatite are hydrophilic. After initial hydration, collagen degrades, especially when exposed to moisture for long periods. The absence of oils means that degradation will proceed without any potential inhibitory effect on bacterial growth or additional stabilization, leading to a straightforward breakdown over time.

Basil EO has a hydrophobic character due to its main components like estragole, eugenol, and eucalyptol, and decreased the water absorption capacity of the A1B sample compared to the R1 sample. Cinnamon EO contains cinnamaldehyde, which has stronger antimicrobial and antioxidant properties than basil EO, which could influence the hydration and degradation pattern. The A1C sample absorbed less water than the one with basil and the control one (R1); this may be because the main component, cinnamaldehyde, can also have the role of cross-linking agent for collagen, forming a more compact structure. The A1C sample absorbed about 31–35 g/g water and AB1 about 32–36 g/g. Regarding the degradation process following the absorption, cinnamon EO slows down the degradation process more effectively than basil EO due to its potent antimicrobial properties. This can lead to prolonged stability of the collagen matrix even in a hydrated environment, though the water absorption might be slightly lower due to the EO’s hydrophobic nature. The combination of both basil and cinnamon EO from sample A1BC, both hydrophobic, is surprising in increasing the water absorption capacity compared to the AB1 and AC1 samples. However, the synergy between these oils could provide a beneficial effect in terms of wound protection due to their combined antimicrobial properties.

Once hydration was achieved, degradation began for all samples. The degradation rate differed across the samples, with the pure collagen and Hap (R1) degrading faster than the samples containing oils. The sample with both oils (sample A1BC) reduces microbial degradation and slows down the breakdown of the dressing material.

### 3.4. Enzymatic Degradation

In the first 8 h, the control sample R1 degraded 29% and the A1B sample degraded 33.23%.

Before 24 h of immersion in collagenase solution, control sample R1, A1B, and A1BC were completely degraded. In our research, the A1C sample, containing cinnamon essential oil rich in cinnamaldehyde, exhibited a delayed degradation process, with 59.90% degradation in the first 24 h and a slower, more consistent degradation thereafter. This behavior suggests that cinnamaldehyde may act similarly to other aldehydes by forming cross-links with collagen, enhancing its resistance to enzymatic degradation. This observation is consistent with the findings of Fathima et al., who reported increased collagen stability due to aldehyde-induced cross-linking [82].

**Figure 6:**
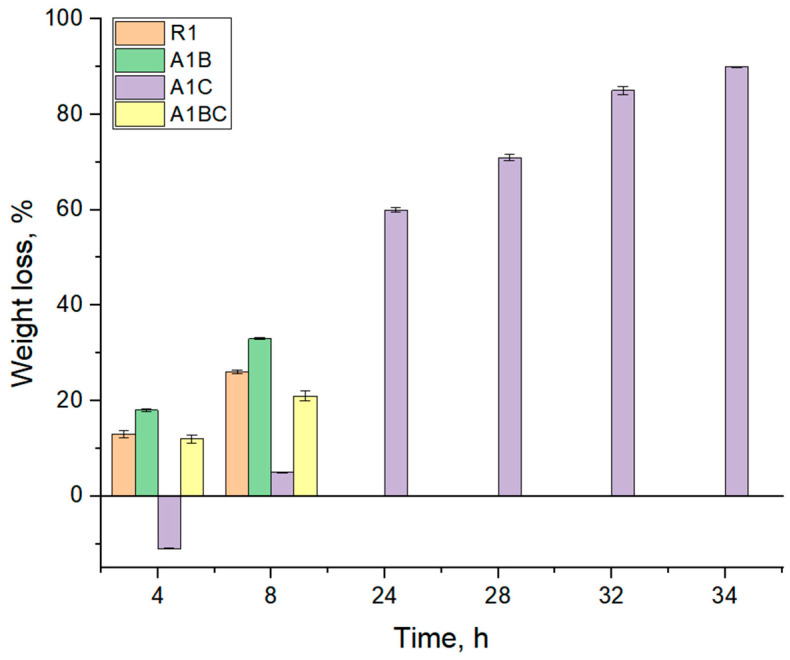
Enzymatic degradation for the investigated samples.

In the first 8 h, the R1 sample degraded by 29% because collagen is highly susceptible to enzymatic degradation by collagenase. Without protective agents (like essential oils), the R1 sample’s collagen structure is quickly broken down. Hydroxyapatite, as a mineral, does not degrade in the same way, but it does not significantly slow collagen degradation. Thus, 29% degradation in the first 8 h directly results from the collagenase acting on the collagen (Figure 6).

The A1B sample degraded 33.23% in the first 8 h, but it does not directly inhibit collagenase activity. Basil EO is hydrophobic, and while it may reduce water absorption to some extent, it does not provide any meaningful protection against enzymatic degradation. As a result, the A1B sample degrades even slightly faster than R1 (33.23% vs. 29%), possibly due to the hydrophobic effect of basil EO, which could make collagen more accessible to enzymatic action in the early stages. While specific studies on the impact of basil essential oil on collagen degradation are limited, research on similar hydrophobic essential oils suggests that they can influence the water absorption dynamics of collagen-based materials, thereby affecting their degradation rates [83]. After 24 h, the R1, A1B, and A1BC samples were completely degraded. By 24 h, the collagenase enzyme has fully broken down the collagen in all three samples. Neither hydroxyapatite nor basil EO (in A1B and A1BC) significantly protects against this enzymatic breakdown. The presence of basil EO alone (A1B) or combined with cinnamon EO (A1BC) does not provide enough structural protection to withstand enzymatic action over a longer period. Despite having both basil and cinnamon EO, the A1BC sample does not resist degradation beyond 24 h. While essential oils may have some antimicrobial and antioxidant properties, they are not collagenase inhibitors, and their hydrophobic nature may expose collagen more easily to enzymatic action once the water absorption phase is complete. The A1C sample contains cinnamon oil, which likely affects water absorption dynamics. Cinnamon oil is hydrophobic, so the sample takes longer to absorb water than R1 and A1B. During the first 4 h, A1C is mainly in the absorption phase, likely due to the barrier created by the oil, which delays collagen exposure to the collagenase enzyme. The degradation process starts after 4 h, being intense in the first 24 h (59.90% degradation). Once water is fully absorbed, collagen becomes more exposed to collagenase, leading to rapid degradation. The hydrophobic barrier provided by the cinnamon oil slows this process initially, but once hydration is complete, collagenase can act on the collagen. The rapid 59.90% degradation in the first 24 h indicates that, despite cinnamon oil’s protective effect against immediate enzymatic action, the collagen breaks down rapidly once the process starts. After 24 h, the degradation process slows down, reaching a constant rate.

This could be due to a couple of reasons: the remaining intact collagen may be more strongly shielded by the residual cinnamon oil, slowing the enzyme’s access, or the breakdown of collagen may leave behind fragments or cross-linked sections that are less accessible to the enzyme. Thus, after the initial rapid degradation, only 31% more of the sample degrades between 24 and 72 h, suggesting that the remaining collagen structure is more resistant to further degradation due to the presence of cinnamon oil [84]. Cinnamon oil, rich in cinnamaldehyde, exhibits antimicrobial, antioxidant, and anti-inflammatory properties. In bioactive dressings, cinnamaldehyde serves as a natural cross-linker for collagen by forming stable covalent bonds with its amino groups [85,86]. This cross-linking reinforces the collagen matrix, making it more resistant to degradation by collagenase, which maintains the structural integrity needed for wound healing. As a result, cinnamaldehyde enhances the mechanical strength and enzymatic stability of collagen-based dressings, supporting tissue repair and improving advanced wound care outcomes. We can conclude that, for this composition, basil EO does not offer significant protection against collagenase-induced degradation. It may even increase the initial rate of degradation slightly due to its hydrophobic properties. However, cinnamon oil delays the initial degradation process by slowing water absorption and may slow down enzymatic activity after prolonged exposure, offering some long-term resistance compared to other samples. However, it is not enough to prevent degradation entirely.

In summary, our findings align with existing literature on the role of aldehydes in enhancing collagen stability through cross-linking mechanisms. The observed degradation behaviors of the A1C and A1B samples can be explained by the interactions of their respective essential oil components with collagen, influencing the material’s resistance to enzymatic degradation [87].

### 3.5. Compression Test

The modulus of elasticity (also known as Young’s modulus) measures a material’s stiffness, which reflects how much it resists deformation under an applied force. This mechanical property is important for wound dressings because it influences the material’s ability to maintain structural integrity while still being flexible enough to conform to the wound area. The results are presented in Table 2. The R1 sample (which contains only collagen and hydroxyapatite) has a modulus of elasticity of 360.70 kPa, indicating moderate stiffness. Collagen, being a biopolymer, contributes to flexibility, while hydroxyapatite, a ceramic material, adds rigidity and support. This balance makes the R1 sample moderately elastic and capable of withstanding compressive forces to some extent. The A1B sample has a higher modulus of elasticity (440.30 kPa) than the R1 sample, indicating that it is stiffer and more deformation resistant. The increase in stiffness is likely due to the inclusion of basil EO, which may interact with the collagen and hydroxyapatite matrix, increasing cross-linking and making the material more rigid. While the increased stiffness provides better mechanical support and may help maintain the structure of the wound dressing over a larger or deeper wound, too much stiffness could reduce flexibility, potentially making the dressing less conformable to the wound. For bioactive wound dressings, some elasticity is important for the patient’s ease of application and comfort. The higher stiffness of the A1B sample might be better suited for wounds that require more structural support. The A1C sample has the lowest modulus of elasticity (299.50 kPa), indicating that it is the least stiff and most flexible. Adding cinnamon oil likely contributes to a more flexible matrix because of its hydrophobic properties and how it interacts with the collagen network, reducing cross-linking density and making the material softer and more pliable. The A1BC sample, containing both basil and cinnamon EO, has a modulus of elasticity of 346.80 kPa, placing it between the R1 sample and the A1C sample in terms of stiffness. The combined effect of basil EO (which tends to increase stiffness) and cinnamon oil (which tends to decrease stiffness) creates a balanced material that is more flexible than A1B but stiffer than A1C. The A1BC sample’s modulus suggests a balance between flexibility and structural support. This intermediate stiffness could make it versatile for various wound types, providing enough support to protect the wound while still being flexible enough to conform to different body contours and provide patient comfort.

**Figure 7:**
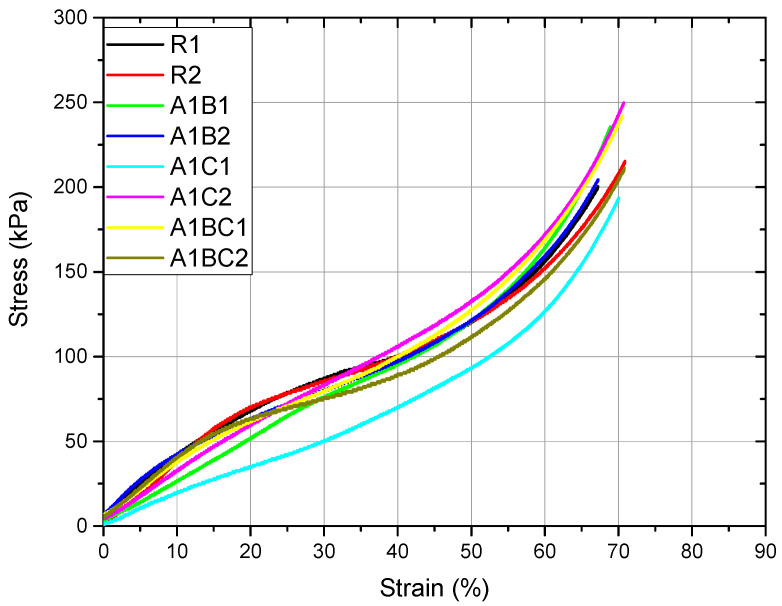
Tension–compression deformation curves.

In general, the behavior is malleable, with significant plastic deformations. The appearance of the curves suggests a gradual compression of the material with increasing stress, similar to porous materials (Figure 7). There are numerous changes in the slopes in different regions of the curve.

The results of the ANOVA test at α = 0.05 indicate that the applied treatment is influencing the modulus of elasticity. The Fisher and Tukey tests indicate an equality of means between R and A1BC. The R1 sample has moderate stiffness and is suitable for general use as a wound dressing that balances flexibility and structural support; the A1B sample has the highest stiffness, offering strong structural support. It may be ideal for larger wounds or wounds that require more stability but might compromise flexibility and comfort in high-mobility areas. A1C is the most flexible, making it ideal for wounds in areas requiring high mobility and flexibility. Still, it might offer less structural support for large or deep wounds, and A1BC has a balanced stiffness, offering moderate flexibility and support, likely the most versatile option for wounds requiring both conformability and mechanical strength. The compression yield strength (Rc0.2) measures the stress at which a material begins to deform plastically. In simpler terms, it is the point where the material will no longer return to its original shape after the force is removed. In the context of bioactive wound dressings, this property is important because it determines how much force the material can withstand before it permanently deforms, which is crucial for maintaining the structural integrity of the dressing when applied to a wound.

The results of the ANOVA test at α = 0.05 indicate that the applied treatment influences the yield strength. The Fisher and Tukey tests suggest the equality of R, A1C, and A1BC means. The treatment applied to A1B reduces the value of the compressive yield strength. The R1 sample has a compression yield strength of 56.00 kPa, indicating that it can withstand a moderate stress level before starting to deform permanently. This value is typical for a collagen-based material reinforced with hydroxyapatite. Collagen provides flexibility, while hydroxyapatite adds rigidity and mechanical support. This compression yield strength is beneficial because it suggests the material can hold its structure when applied to a wound without breaking down or deforming too easily. It is a good balance for use in areas requiring moderate mechanical stability while allowing some flexibility. The A1B sample has a lower compression yield strength (46.06 kPa) than the R1 sample. Adding basil EO likely reduces the overall structural integrity, making the sample less resistant to compression forces. Basil EO’s hydrophobic and flexible nature may reduce the cross-linking between collagen and hydroxyapatite, leading to a material that deforms more easily under pressure. The lower yield strength means the A1B sample is less resistant to compressive forces and may deform more easily when applied to a wound. This could be a disadvantage when the dressing needs to maintain its shape for longer or under more pressure. However, this could still be suitable for wounds in low-pressure areas or where flexibility is more important than structural integrity. The A1C sample has a compression yield strength of 56.61 kPa, which is very similar to that of the R1 sample (56.00 kPa). This suggests that adding cinnamon oil does not significantly alter the material’s resistance to compressive forces compared to the control. Cinnamon oil might contribute to slightly better cross-linking within the collagen matrix, helping to maintain the mechanical integrity like the R1 sample. The A1C sample maintains a balance between flexibility and structural support, with a yield strength that indicates it can withstand moderate pressure without deforming. This makes it well-suited for wounds requiring mechanical support without sacrificing flexibility. The A1BC sample has the highest compression yield strength (56.82 kPa) among all the samples, though it is still quite close to R1 and A1C. The combination of basil and cinnamon EO provides an enhanced interaction within the collagen–hydroxyapatite matrix, resulting in a material that can withstand slightly higher compressive forces before yielding. Despite reducing the yield strength in A1B, basil EO may interact synergistically with cinnamon oil in this sample to improve mechanical stability. The higher yield strength of A1BC means it can resist deformation more effectively than the other samples, making it the most structurally stable dressing. This could be beneficial in applications where the dressing needs to maintain its shape under stress, such as wounds that experience higher pressure or friction. The balance of oils also ensures that the material remains bioactive and flexible while providing superior mechanical stability. The control sample has moderate compression yield strength, offering balanced mechanical support and flexibility and suitable for a wide range of wounds, especially where moderate structural support is needed. This makes it a good option for wounds that need a balance of support and flexibility. The combination of basil and cinnamon EO results in the highest compression yield strength, indicating this sample can withstand the most pressure without deformation. This would be ideal for wounds in high-pressure areas or those requiring excellent mechanical stability.

### 3.6. Shore C Hardness

**Figure 8:**
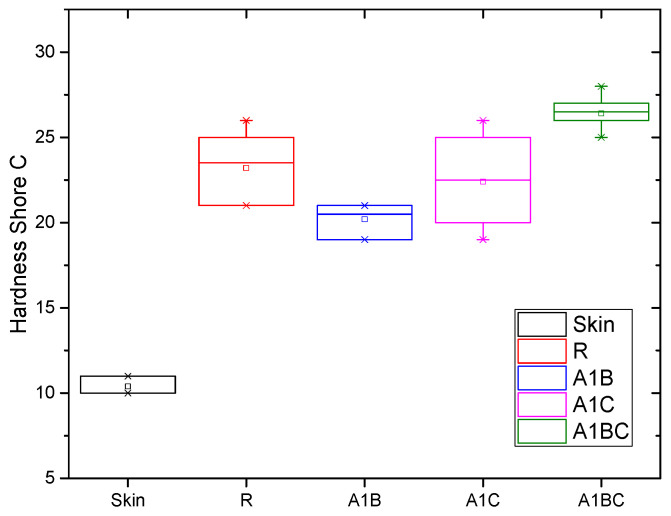
Comparisons between the Shore C hardness of the experimental samples.

The Shore C hardness test measures the hardness of a material, specifically its resistance to indentation. In bioactive wound dressings, hardness is an important factor because it affects the material’s durability, flexibility, and how comfortably it conforms to the skin. A comparison of the Shore C hardness of the experimental samples and human skin (which had a Shore C hardness determinate of 10.4) gives insight into how each sample might perform in wound care (Figure 8).

The skin is relatively soft with a Shore C hardness of 10.4, which provides flexibility and adaptability to body movement. Wound dressings should ideally have some degree of softness to conform to the skin and provide comfort while being durable enough to protect the wound. The Shore C hardness of the wound dressing should not be too high compared to the skin, as this could lead to discomfort, irritation, or poor adherence to the wound area [88]. The R1 sample is significantly harder than human skin, with a Shore C hardness of 23.2. The combination of collagen (a relatively soft, flexible biopolymer) and hydroxyapatite (a rigid, ceramic-like material) results in a harder composite. While this hardness provides durability and structural support, it may be less comfortable compared to softer materials and could feel stiff when applied to flexible areas of the body. This hardness level makes R1 suitable for wounds requiring mechanical protection and structural stability. Still, it might be less ideal for wounds on highly mobile body parts (like joints), where a more flexible dressing would be preferred. The A1B sample, with a Shore C hardness of 20.2, is softer than the R1 sample. Adding basil EO, which has a hydrophobic and flexible nature, likely reduces the overall hardness of the material by decreasing the rigidity of the collagen–hydroxyapatite matrix. This lower hardness means the sample is more flexible and closer in hardness to human skin than R1. This sample’s hardness suggests that it would be more comfortable for the patient, as it is softer and could better conform to the wound surface. However, this comes at the cost of reduced mechanical strength compared to the R1 sample, meaning it might not offer the same level of protection in wounds that need more support. The A1C sample has a Shore C hardness of 22.4, slightly softer than R1 but still relatively close in hardness. Including cinnamon oil seems to provide some flexibility without drastically reducing hardness, likely due to its impact on the overall structure of the collagen matrix. The A1C sample remains slightly harder than human skin but offers a better structural integrity and flexibility balance than R1. With a hardness close to R1 but slightly softer, A1C could offer a more comfortable application without sacrificing durability. It is suited for wounds that need moderate protection but would benefit from a slightly softer dressing to improve conformability. The A1BC sample has the highest Shore C hardness at 26.4, making it the hardest of all the samples. The combination of basil and cinnamon EO has led to a more rigid material than the A1B and A1C samples. This suggests that the two oils, when combined, increase cross-linking or provide structural support within the collagen–hydroxyapatite matrix, resulting in a more rigid material. While this sample offers the greatest mechanical strength and durability, it may be too stiff for wounds in areas where flexibility and comfort are critical. Its higher hardness could limit its ability to conform to wound shapes, especially in areas with a lot of movement, potentially causing discomfort to the patient. Softer materials (like A1B and A1C) are closer in hardness to human skin and are likely to be more comfortable for the patient. These materials could better conform to the wound, especially in areas with a lot of movement or curvature. They are more suitable for superficial wounds where flexibility and patient comfort are priorities. More rigid materials (like R1 and A1BC) provide better mechanical protection.

In conclusion, the Shore C hardness results suggest that different experimental samples will have varying performance characteristics based on their composition, with softer samples being more patient-friendly and harder samples providing more mechanical protection. The ANOVA performed on the Shore C hardness values of the experimental samples at α = 0.05 indicates statistically significant differences, which indicates that the applied treatment is an influencing factor for hardness. Tukey and Fisher tests evaluated the differences between the means and suggested an equality between the A1C–R means.

### 3.7. Roughness

Regarding tissue regeneration and antibacterial applications, their roughness can greatly impact the biological performance of materials based on collagen, hydroxyapatite, and essential oils. For example, depending on the surface roughness, composite materials, including collagen and hydroxyapatite, such as those improved with essential oils, might influence cell adhesion, proliferation, and antibacterial activity. Because of their greater surface area, rougher surfaces can encourage better cell contact, particularly for osteoblasts, and contribute to bacterial inhibition [89]. Studies on hydroxyapatite–collagen composites laced with polydopamine have shown that these materials can control osteoclast activity, which is good for bone regeneration [90,91]. Roughness is a key surface characteristic that regulates how cells like osteoclasts and osteoblasts act on these materials. Additionally, other research investigates how surface roughness can boost a material’s antibacterial qualities and biological compatibility when paired with essential oils or nanoherbal extracts [92]. The surface roughness of wound dressing materials is crucial in their interaction with wound tissues, fluid absorption, and overall healing properties. Roughness can affect how well cells adhere to the surface, influence fluid management, and modify the antimicrobial properties of the dressing [89].

**Figure 9:**
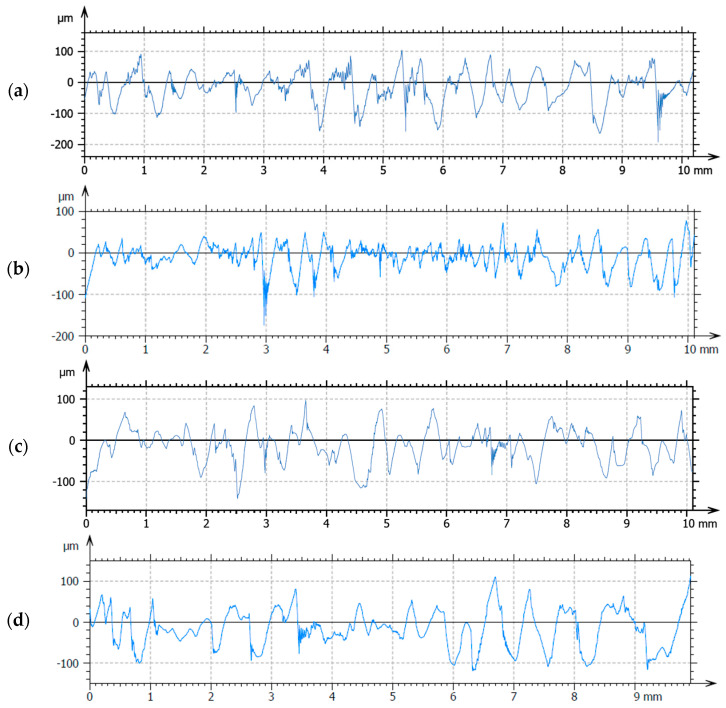
Roughness surface properties for composite sponges: (**a**) R1, (**b**) A1C, (**c**) A1B, and (**d**) A1BC.

Figure 9 presents the roughness profiles for the investigated samples. The reference sample (R1) containing only collagen and hydroxyapatite has a relatively high surface roughness (10.5933 µm) due to collagen’s porous and fibrous structure and the dispersion of hydroxyapatite particles within the matrix. Microscopically, collagen fibers tend to form a scaffold-like, interwoven structure, and hydroxyapatite particles appear as small granules or crystals, which add to the surface texture. Higher roughness in sample R1 could be beneficial for cell adhesion and proliferation, which are essential for tissue regeneration and healing [93]. A rougher surface provides more surface area for biological interactions, such as the attachment of proteins, fibroblasts, and keratinocytes. This is especially important for wound healing, as it encourages cell migration and growth. The rough, porous structure also helps with fluid absorption, which is important for managing wound exudate. Adding basil EO decreased the surface roughness of sample A1B at 8.08084 µm compared to the reference sample. Basil EO contains hydrophobic compounds (e.g., linalool, eugenol), which could partially coat the surface of the collagen–hydroxyapatite matrix, leading to a smoother surface. A reduction in roughness could mean slightly less cell attachment compared to the reference sample [91]. However, the presence of basil EO’s antimicrobial properties compensates for this by protecting against infection. The smoother surface may also reduce excess fluid absorption, as the oil could act as a barrier, making the sample more hydrophobic [86]. Also, a smoother surface may reduce friction, potentially minimizing shear stress when the dressing is applied to the wound, which can benefit patient comfort and reduce further tissue damage. Similar to A1B, adding cinnamon oil in the A1C sample will also reduce the roughness at 8.8274 µm compared to R1, which has an Ra of 10.5933. Cinnamon oil, containing cinnamaldehyde and other hydrophobic compounds, can create a smoother surface by filling gaps in the collagen–hydroxyapatite matrix. While the surface might not be as smooth as the basil EO sample, it still has a lower roughness than the reference due to the hydrophobic coating effects of the essential oil. Cinnamon oil’s slight reduction in roughness could result in moderate cell attachment. The antimicrobial properties of cinnamon oil would, again, enhance the dressing’s infection control capabilities. The moderately smooth surface, while still slightly textured, could help balance moisture retention and exudate management [92]. The slight texture may still allow some exudate absorption while preventing excessive moisture from accumulating on the wound. The A1BC sample has a medium roughness of 9.1363 µm, although combining the two oils was expected to generate the smoothest surface. The smooth surface from sample A1C will likely resist bacterial colonization and promote a cleaner wound environment, though it might not be ideal for wounds needing exudate control.

### 3.8. Antimicrobial Activity

**Figure 10:**
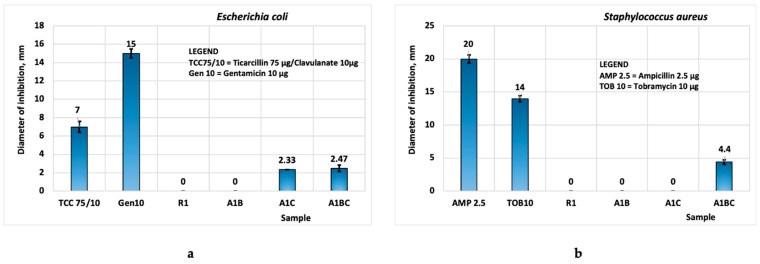
Antimicrobial activities of biomaterials tested: (**a**) local antibacterial effect of biomaterials for *Escherichia coli*; (**b**) local antibacterial activity of biomaterial for *Staphylococcus aureus*.

The antimicrobial studies conducted on the two bacterial strains showed the following: biomaterials R1 and A1B do not exhibit antibacterial activity against the tested bacteria. Both biomaterials A1C and A1BC exhibit antimicrobial activity for *Escherichia coli* (Figure 10a), activity attributed to specific antimicrobial compounds from cinnamon EO and basil EO [38,59,94]. The biomaterial A1C (with cinnamon EO) (Figure 10b) does not exhibit antimicrobial properties against *Staphylococcus aureus*, likely due to the lower content of essential oil used. The composite biomaterial A1BC exhibits a local antimicrobial effect against *Staphylococcus aureus*, an effect attributed to specific compounds from basil EO, such as sabinene, myrcene, and linalool [38,59,94].

In conclusion, the most promising biomaterial with local antimicrobial activity is A1BC, containing 1% basil EO and 1% cinnamon EO, for both *Escherichia coli* and *Staphylococcus aureus.* This may be due to the synergic effect of the combination of basil and cinnamon oils, their main components acting together and providing antimicrobial activity.

## 4. Conclusions

The experimental bioactive wound dressings developed in this study, combining collagen, hydroxyapatite, and essential oils of basil and cinnamon, demonstrated distinct properties tailored to specific wound-healing needs. The inclusion of basil and cinnamon essential oils enhanced antimicrobial activity, mechanical strength, and degradation resistance, while varying porosity and flexibility allowed for targeted applications. Notably, the A1BC composition showed the highest antimicrobial efficacy and mechanical resistance, making it ideal for high-stress wounds, whereas R1 and A1B offered greater flexibility for less demanding scenarios.

These findings underscore the versatility and potential of these bioactive dressings for diverse wound-healing applications. Future research should focus on validating these results in animal models and clinical settings to establish their broader applicability and effectiveness. One limitation of our study is related to the use of *Escherichia coli* (*E. coli*) as a Gram-positive bacterial model for antimicrobial testing, even though it is not a primary target for wound infections, due to its high prevalence in hospital environments.

## Figures and Tables

**Table 1:** Compositions of the designed composite gels [wt.%].

Sample	Coll (wt.%)	HAp %	Basil EO %	Cinnamon EO %
R1	1	1	0	0
A1B	1	1	1	0
A1C	1	1	0	1
A1BC	1	1	1	1

**Table 2:** Modulus of elasticity and compression yield strength results for the experimental samples.

Samples	R	A1B	A1C	A1BC
E[kPa]Average	360.70	440.30	299.50	346.80
Standard deviation	22.03	5.08	7.03	17.93
Rc0.2[kPa]Average	56.00	46.06	56.61	56.82
Standard deviation	3.55	4.78	3.17	0.38

## Data Availability

The original contributions presented in the study are included in the article, further inquiries can be directed to the corresponding author.

## References

[1] Yousefian F., Hesari R., Jensen T., Obagi S., Rgeai A., Damiani G., Bunick C.G., Grada A. (2023). Antimicrobial Wound Dressings: A Concise Review for Clinicians. Antibiotics.

[2] Selem E., Mekky A.F., Hassanein W.A., Reda F.M., Selim Y.A. (2022). Antibacterial and Antibiofilm Effects of Silver Nanoparticles against the Uropathogen Escherichia Coli U12. Saudi J. Biol. Sci..

[3] Gheorghita D., Robu A., Antoniac A., Antoniac I., Ditu L.M., Raiciu A.-D., Tomescu J., Grosu E., Saceleanu A. (2022). In Vitro Antibacterial Activity of Some Plant Essential Oils against Four Different Microbial Strains. Appl. Sci..

[4] Gheorghiță D., Moldovan H., Robu A., Bița A.-I., Grosu E., Antoniac A., Corneschi I., Antoniac I., Bodog A.D., Băcilă C.I. (2023). Chitosan-Based Biomaterials for Hemostatic Applications: A Review of Recent Advances. Int. J. Mol. Sci..

[5] Gheorghita D., Grosu E., Robu A., Ditu L.M., Deleanu I.M., Gradisteanu Pircalabioru G., Raiciu A.D., Bita A.I., Antoniac A., Antoniac V.I. (2022). Essential Oils as Antimicrobial Active Substances in Wound Dressings. Materials.

[6] Parenteau-Bareil R., Gauvin R., Berthod F. (2010). Collagen-Based Biomaterials for Tissue Engineering Applications. Materials.

[7] Albu M.G., Vladkova T.G., Ivanova I.A., Shalaby A.S.A., Moskova-Doumanova V.S., Staneva A.D., Dimitriev Y.B., Kostadinova A.S., Topouzova-Hristova T.I. (2016). Preparation and Biological Activity of New Collagen Composites, Part I: Collagen/Zinc Titanate Nanocomposites. Appl. Biochem. Biotechnol..

[8] Boateng J.S., Matthews K.H., Stevens H.N.E., Eccleston G.M. (2008). Wound Healing Dressings and Drug Delivery Systems: A Review. J. Pharm. Sci..

[9] Pitterou I., Kalogeropoulou F., Tzani A., Tsiantas K., Gatou M.A., Pavlatou E., Batrinou A., Fountzoula C., Kriebardis A., Zoumpoulakis P. (2024). Development of Alginate Hydrogels Incorporating Essential Oils Loaded in Chitosan Nanoparticles for Biomedical Applications. Molecules.

[10] Pulvirenti A., Boccia A.C., Constantin C., Surcel M., Munteanu A., Peteu V.E., Neagu M. (2024). Single-Component Starch-Based Hydrogels for Therapeutic Delivery. Molecules.

[11] Ding X., Yang B., Hou Z. (2024). In Situ Crosslinked Biodegradable Hydrogels Based on Poly(Ethylene Glycol) and Poly(ε-Lysine) for Medical Application. Molecules.

[12] Ansari M., Darvishi A. (2024). A Review of the Current State of Natural Biomaterials in Wound Healing Applications. Front. Bioeng. Biotechnol..

[13] Robu A., Antoniac A., Ciocoiu R., Grosu E., Rau J.V., Fosca M., Krasnyuk I.I., Pircalabioru G.G., Manescu (Paltanea) V., Antoniac I. (2022). Effect of the Antimicrobial Agents Peppermint Essential Oil and Silver Nanoparticles on Bone Cement Properties. Biomimetics.

[14] Pavlík V., Sobotka L., Pejchal J., Čepa M., Nešporová K., Arenbergerová M., Mrózková A., Velebný V. (2021). Silver Distribution in Chronic Wounds and the Healing Dynamics of Chronic Wounds Treated with Dressings Containing Silver and Octenidine. FASEB J..

[15] Thomas A., Bankar N., Nagore D., Kothapalli L., Chitlange S. (2022). Herbal Oils for Treatment of Chronic and Diabetic Wounds: A Systematic Review. Curr. Diabetes Rev..

[16] do Nascimento A.S., Tamiasso R.S.S., Morais S.F.M., Rizzo Gnatta J., Turrini R.N.T., Calache A.L.S.C., de Brito Poveda V. (2022). Essential Oils for Healing and/or Preventing Infection of Surgical Wounds: A Systematic Review. Rev. Esc. Enferm. USP.

[17] Alven S., Peter S., Aderibigbe B.A. (2022). Polymer-Based Hydrogels Enriched with Essential Oils: A Promising Approach for the Treatment of Infected Wounds. Polymers.

[18] Vasile B.S., Oprea O., Voicu G., Ficai A., Andronescu E., Teodorescu A., Holban A. (2014). Synthesis and Characterization of a Novel Controlled Release Zinc Oxide/Gentamicin–Chitosan Composite with Potential Applications in Wounds Care. Int. J. Pharm..

[19] Homaeigohar S., Boccaccini A.R. (2020). Antibacterial Biohybrid Nanofibers for Wound Dressings. Acta Biomater..

[20] Wang X., Chang J., Wu C. (2018). Bioactive Inorganic/Organic Nanocomposites for Wound Healing. Appl. Mater. Today.

[21] Costa N.N., de Faria Lopes L., Ferreira D.F., de Prado E.M.L., Severi J.A., Resende J.A., de Paula Careta F., Ferreira M.C.P., Carreira L.G., de Souza S.O.L. (2020). Polymeric Films Containing Pomegranate Peel Extract Based on PVA/Starch/PAA Blends for Use as Wound Dressing: In Vitro Analysis and Physicochemical Evaluation. Mater. Sci. Eng. C.

[22] Tudoroiu E.E., Dinu-Pîrvu C.E., Kaya M.G.A., Popa L., Anuța V., Prisada R.M., Ghica M.V. (2021). An Overview of Cellulose Derivatives-Based Dressings for Wound-Healing Management. Pharmaceuticals.

[23] Koehler J., Brandl F.P., Goepferich A.M. (2018). Hydrogel Wound Dressings for Bioactive Treatment of Acute and Chronic Wounds. Eur. Polym. J..

[24] Santamaria N., Woo K., Beeckman D., Alves P., Cullen B., Gefen A., Lázaro-Martínez J.L., Lev-Tov H., Najafi B., Sharpe A. (2023). Clinical Performance Characteristics for Bordered Foam Dressings in the Treatment of Complex Wounds: An International Wound Dressing Technology Expert Panel Review. Int. Wound J..

[25] Zare-Gachi M., Daemi H., Mohammadi J., Baei P., Bazgir F., Hosseini-Salekdeh S., Baharvand H. (2020). Improving Anti-Hemolytic, Antibacterial and Wound Healing Properties of Alginate Fibrous Wound Dressings by Exchanging Counter-Cation for Infected Full-Thickness Skin Wounds. Mater. Sci. Eng. C.

[26] De Luca I., Pedram P., Moeini A., Cerruti P., Peluso G., Di Salle A., Germann N. (2021). Nanotechnology Development for Formulating Essential Oils in Wound Dressing Materials to Promote the Wound-Healing Process: A Review. Appl. Sci..

[27] Rezvani Ghomi E., Khalili S., Nouri Khorasani S., Esmaeely Neisiany R., Ramakrishna S. (2019). Wound Dressings: Current Advances and Future Directions. J. Appl. Polym. Sci..

[28] Dursun N., Liman N., Özyazgan İ., Güneş I., Saraymen R. (2003). Role of Thymus Oil in Burn Wound Healing. J. Burn. Care Rehabil..

[29] Khansa I., Schoenbrunner A.R., Kraft C.T., Janis J.E. (2019). Silver in Wound Care—Friend or Foe?: A Comprehensive Review. Plast. Reconstr. Surg. Glob. Open.

[30] Lee K., Lee S. (2020). Electrospun Nanofibrous Membranes with Essential Oils for Wound Dressing Applications. Fibers Polym..

[31] Maver T., Kurečič M., Maja Smrke D., Stana Kleinschek K., Maver U. (2019). Plant-Derived Medicines with Potential Use in Wound Treatment. Herbal Medicine.

[32] Nešporová K., Pavlík V., Šafránková B., Vágnerová H., Odráška P., Žídek O., Císařová N., Skoroplyas S., Kubala L., Velebný V. (2020). Effects of Wound Dressings Containing Silver on Skin and Immune Cells. Sci. Rep..

[33] Yammine J., Chihib N.-E., Gharsallaoui A., Dumas E., Ismail A., Karam L. (2022). Essential Oils and Their Active Components Applied as: Free, Encapsulated and in Hurdle Technology to Fight Microbial Contaminations. A Review. Heliyon.

[34] Nazzaro F., Fratianni F., De Martino L., Coppola R., De Feo V. (2013). Effect of Essential Oils on Pathogenic Bacteria. Pharmaceuticals.

[35] Aelenei P., Miron A., Trifan A., Bujor A., Gille E., Aprotosoaie A. (2016). Essential Oils and Their Components as Modulators of Antibiotic Activity against Gram-Negative Bacteria. Medicines.

[36] Yang S.-K., Tan N.-P., Chong C.-W., Abushelaibi A., Lim S.-H.-E., Lai K.-S. (2021). The Missing Piece: Recent Approaches Investigating the Antimicrobial Mode of Action of Essential Oils. Evol. Bioinform..

[37] Ojah E.O. (2020). Exploring Essential Oils as Prospective Therapy against the Ravaging Coronavirus (SARS-CoV-2). Iberoam. J. Med..

[38] Fachriyah E., Wibawa P.J., Awaliyah A. (2020). Antibacterial Activity of Basil Oil (*Ocimum basilicum* L.) and Basil Oil Nanoemulsion. J. Phys. Conf. Ser..

[39] Brdjanin S., Bogdanovic N., Kolundzic M., Milenkovic M., Golic N., Kojic M., Kundakovic T. (2015). Antimicrobial Activity of Oregano (*Origanum vulgare* L.): And Basil (*Ocimum basilicum* L.): Extracts. Adv. Technol..

[40] Al Abbasy D.W., Pathare N., Al-Sabahi J.N., Khan S.A. (2015). Chemical Composition and Antibacterial Activity of Essential Oil Isolated from Omani Basil (*Ocimum basilicum* Linn.). Asian Pac. J. Trop. Dis..

[41] Kalkhorani N.M., Dadgar M., Rezaee M.B., Mahboubi A., HeroAbad F. (2017). Essential Oils Composition of Ocimum Basilicum Var. Purpurascens FromDifferent Ecological Zone in Iran and Antimicrobial Activity against Different Bacterial Species. J. Med. Plants By-Prod..

[42] Miguel M.G. (2010). Antioxidant and Anti-Inflammatory Activities of Essential Oils: A Short Review. Molecules.

[43] Li C., Zhang C., Chen X., Cui H., Lin L. (2022). The Interference Mechanism of Basil Essential Oil on the Cell Membrane Barrier and Respiratory Metabolism of Listeria Monocytogenes. Front. Microbiol..

[44] Kamelnia E., Mohebbati R., Kamelnia R., El-Seedi H.R., Boskabady M.H. (2023). Anti-Inflammatory, Immunomodulatory and Anti-Oxidant Effects of *Ocimum basilicum* L. and Its Main Constituents: A Review. Iran. J. Basic. Med. Sci..

[45] Hadi M., Moghtadaei-Khorasgani E., Etesamnia M.H. (2021). The Influence of Basil Seed Hydroethanolic Extract on the Skin Wound Healing in Diabetic Male Rats. World’s Vet. J..

[46] Antonescu I.A., Antonescu A., Miere F., Fritea L., Teușdea A.C., Vicaș L., Vicaș S.I., Brihan I., Domuța M., Zdrinca M. (2021). Evaluation of Wound Healing Potential of Novel Hydrogel Based on Ocimum Basilicum and Trifolium Pratense Extracts. Processes.

[47] Mohammadi L., Hassanzadeh Khankahdani H., Tanaka F., Tanaka F. (2020). Effect of Aloe Vera Gel Combined with Basil (*Ocimum basilicum* L.) Essential Oil as a Natural Coating on Maintaining Post-Harvest Quality of Peach (*Prunus persica* L.) during Storage. IOP Conf. Ser. Earth Environ. Sci..

[48] Bejeshk M.A., Aminizadeh A.H., Rajizadeh M.A., Khaksari M., Lashkarizadeh M., Shahrokhi N., Zahedi M.J., Azimi M. (2022). The Effect of Combining Basil Seeds and Gum Arabic on the Healing Process of Experimental Acetic Acid-Induced Ulcerative Colitis in Rats. J. Tradit. Complement. Med..

[49] Antonescu (Mintas) A.-I., Miere (Groza) F., Fritea L., Ganea M., Zdrinca M., Dobjanschi L., Antonescu A., Vicas S.I., Bodog F., Sindhu R.K. (2021). Perspectives on the Combined Effects of *Ocimum basilicum* and *Trifolium pratense* Extracts in Terms of Phytochemical Profile and Pharmacological Effects. Plants.

[50] Zhakipbekov K., Turgumbayeva A., Akhelova S., Bekmuratova K., Blinova O., Utegenova G., Shertaeva K., Sadykov N., Tastambek K., Saginbazarova A. (2024). Antimicrobial and Other Pharmacological Properties of *Ocimum basilicum*, Lamiaceae. Molecules.

[51] Doyle A.A., Stephens J.C. (2019). A Review of Cinnamaldehyde and Its Derivatives as Antibacterial Agents. Fitoterapia.

[52] Vasconcelos N.G., Croda J., Simionatto S. (2018). Antibacterial Mechanisms of Cinnamon and Its Constituents: A Review. Microb. Pathog..

[53] Chang S.-T., Chen P.-F., Chang S.-C. (2001). Antibacterial Activity of Leaf Essential Oils and Their Constituents from Cinnamomum Osmophloeum. J. Ethnopharmacol..

[54] Raeisi M., Tajik H., Yarahmadi A., Sanginabadi S. (2015). Antimicrobial Effect of Cinnamon Essential Oil Against Escherichia Coli and Staphylococcus Aureus. Health Scope.

[55] Mayaud L., Carricajo A., Zhiri A., Aubert G. (2008). Comparison of Bacteriostatic and Bactericidal Activity of 13 Essential Oils against Strains with Varying Sensitivity to Antibiotics. Lett. Appl. Microbiol..

[56] Noura E., Kamilia A.M.A. (2021). The Antibacterial Activity of Nano-Encapsulated Basil and Cinnamon Essential Oils against Certain Multidrug-Resistant Bacteria Recovered from Infected Wounds. Nov. Res. Microbiol. J..

[57] López P., Sánchez C., Batlle R., Nerín C. (2005). Solid- and Vapor-Phase Antimicrobial Activities of Six Essential Oils: Susceptibility of Selected Foodborne Bacterial and Fungal Strains. J. Agric. Food Chem..

[58] El Atki Y., Aouam I., El Kamari F., Taroq A., Nayme K., Timinouni M., Lyoussi B., Abdellaoui A. (2019). Antibacterial Activity of Cinnamon Essential Oils and Their Synergistic Potential with Antibiotics. J. Adv. Pharm. Technol. Res..

[59] Buriti B.M.A.D.B., Figueiredo P.L.B., Passos M.F., da Silva J.K.R. (2024). Polymer-Based Wound Dressings Loaded with Essential Oil for the Treatment of Wounds: A Review. Pharmaceuticals.

[60] Vranceanu M.D., Antoniac I.V., Miculescu F., Vrânceanu M.D., Şaban R., Antoniac I., Albu M. (2012). Development and Characterization of Novel Porous Collagen Based Biocomposite for Bone Tissue Regeneration. Bull. Ser. B.

[61] Popescu F., Titorencu I., Albu Kaya M., Miculescu F., Tutuianu R., Coman A.E., Danila E., Marin M.M., Ancuta D.-L., Coman C. (2024). Development of Innovative Biocomposites with Collagen, Keratin and Hydroxyapatite for Bone Tissue Engineering. Biomimetics.

[62] Mederle N., Marin S., Marin M.M., Danila E., Mederle O., Albu Kaya M.G., Ghica M.V. (2016). Innovative Biomaterials Based on Collagen-Hydroxyapatite and Doxycycline for Bone Regeneration. Adv. Mater. Sci. Eng..

[63] Yuan Y., Hays M.P., Hardwidge P.R., Kim J. (2017). Surface Characteristics Influencing Bacterial Adhesion to Polymeric Substrates. RSC Adv..

[64] Zhang X., Wang L., Levänen E. (2013). Superhydrophobic Surfaces for the Reduction of Bacterial Adhesion. RSC Adv..

[65] Stallard C.P., McDonnell K.A., Onayemi O.D., O’Gara J.P., Dowling D.P. (2012). Evaluation of Protein Adsorption on Atmospheric Plasma Deposited Coatings Exhibiting Superhydrophilic to Superhydrophobic Properties. Biointerphases.

[66] Seyed Ahmadi S.G., Farahpour M.R., Hamishehkar H. (2019). Topical Application of Cinnamon Verum Essential Oil Accelerates Infected Wound Healing Process by Increasing Tissue Antioxidant Capacity and Keratin Biosynthesis. Kaohsiung J. Med. Sci..

[67] Breijyeh Z., Karaman R. (2024). Antibacterial Activity of Medicinal Plants and Their Role in Wound Healing. Futur. J. Pharm. Sci..

[68] Marcut L., Manescu V., Antoniac A., Paltanea G., Robu A., Mohan A.G., Grosu E., Corneschi I., Bodog A.D. (2023). Antimicrobial Solutions for Endotracheal Tubes in Prevention of Ventilator-Associated Pneumonia. Materials.

[69] Albu M.G. (2011). Collagen Gels and Matrices for Biomedical Applications: The Obtaining and Characterization of Collagen-Based Biomaterials as Support for Local Release.

[70] (2022). International Standard.

[71] (2008). Plastics–Determination of Water Absorption.

[72] (2015). International Standard.

[73] Standard Test Method for Rubber Property-Durometer Hardness D2240-00. https://www.plantech.com/wp-content/uploads/2017/05/ASTM-D2240-Durometer-Hardness.pdf.

[74] Chang M.C., Tanaka J. (2002). FT-IR Study for Hydroxyapatite/Collagen Nanocomposite Cross-Linked by Glutaraldehyde. Biomaterials.

[75] Takallu S., Mirzaei E., Azadi A., Karimizade A., Tavakol S. (2019). Plate-shape Carbonated Hydroxyapatite/Collagen Nanocomposite Hydrogel via in Situ Mineralization of Hydroxyapatite Concurrent with Gelation of Collagen at PH = 7.4 and 37 °C. J. Biomed. Mater. Res. B Appl. Biomater..

[76] Antoniac I.V., Antoniac A., Vasile E., Tecu C., Fosca M., Yankova V.G., Rau J.V. (2021). In Vitro Characterization of Novel Nanostructured Collagen-Hydroxyapatite Composite Scaffolds Doped with Magnesium with Improved Biodegradation Rate for Hard Tissue Regeneration. Bioact. Mater..

[77] Mendes L.C., Ribeiro G.L., Marques R.C. (2012). In Situ Hydroxyapatite Synthesis: Influence of Collagen on Its Structural and Morphological Characteristic. Mater. Sci. Appl..

[78] Plepis A.M.G., Rodrigues F.T., Martins V.C.A. (2010). Porcine Skin as a Source of Biodegradable Matrices: Alkaline Treatment and Glutaraldehyde Crosslinking. Polímeros.

[79] Cheng X., Shao Z., Li C., Yu L., Raja M.A., Liu C. (2017). Isolation, Characterization and Evaluation of Collagen from Jellyfish Rhopilema Esculentum Kishinouye for Use in Hemostatic Applications. PLoS ONE.

[80] Chen R., Huang C., Ke Q., He C., Wang H., Mo X. (2010). Preparation and Characterization of Coaxial Electrospun Thermoplastic Polyurethane/Collagen Compound Nanofibers for Tissue Engineering Applications. Colloids Surf. B Biointerfaces.

[81] Szychlinska M.A., Calabrese G., Ravalli S., Dolcimascolo A., Castrogiovanni P., Fabbi C., Puglisi C., Lauretta G., Di Rosa M., Castorina A. (2020). Evaluation of a Cell-Free Collagen Type i-Based Scaffold for Articular Cartilage Regeneration in an Orthotopic Rat Model. Materials.

[82] Fathima N.N., Madhan B., Rao J.R., Nair B.U., Ramasami T. (2004). Interaction of Aldehydes with Collagen: Effect on Thermal, Enzymatic and Conformational Stability. Int. J. Biol. Macromol..

[83] Fraternale D., Flamini G., Ascrizzi R. (2019). In Vitro Anticollagenase and Antielastase Activities of Essential Oil of Helichrysum Italicum Subsp. Italicum (Roth) G. Don. J. Med. Food.

[84] Zhang S., Sun X., Lei Y., Sun B., Xie P., Liu X. (2022). Effects of Chitosan/Collagen Peptides/Cinnamon Bark Essential Oil Composite Coating on the Quality of Dry-Aged Beef. Foods.

[85] Takasao N., Tsuji-Naito K., Ishikura S., Tamura A., Akagawa M. (2012). Cinnamon Extract Promotes Type i Collagen Biosynthesis via Activation of IGF-I Signaling in Human Dermal Fibroblasts. J. Agric. Food Chem..

[86] Fu H., Park J., Pei D. (2002). Peptidyl Aldehydes as Reversible Covalent Inhibitors of Protein Tyrosine Phosphatases. Biochemistry.

[87] Berechet M.D., Gaidau C., Miletic A., Pilic B., Râpă M., Stanca M., Ditu L.M., Constantinescu R., Lazea-Stoyanova A. (2020). Bioactive Properties of Nanofibres Based on Concentrated Collagen Hydrolysate Loaded with Thyme and Oregano Essential Oils. Materials.

[88] Kalra A., Lowe A. (2016). Mechanical Behaviour of Skin: A Review. J. Mater. Sci. Eng..

[89] Eswaramoorthy N., McKenzie D.R. (2017). Plasma Treatments of Dressings for Wound Healing: A Review. Biophys. Rev..

[90] Ficai A., Andronescu E., Voicu G., Ficai D. (2011). Advances in Collagen/Hydroxyapatite Composite Materials. Advances in Composite Materials for Medicine and Nanotechnology.

[91] Wang L., Wu T.-H., Hu X., Liu J., Wu D., Miguez P.A., Wright J.T., Zhang S., Chi J.-T., Tseng H.C. (2021). Biomimetic Polydopamine-Laced Hydroxyapatite Collagen Material Orients Osteoclast Behavior to an Anti-Resorptive Pattern without Compromising Osteoclasts’ Coupling to Osteoblasts. Biomater. Sci..

[92] Moharam L.M., Sadony D.M., Adel M.M., Montasser K. (2021). Evaluation of Surface Roughness and Vickers Microhardness of Various Nano-Herbal Extracts on Demineralized Dentin and Their Bactericidal Efficacy with 970-Nm Wavelength Diode Laser Irradiation. Bull. Natl. Res. Cent..

[93] Miwa M., Nakajima A., Fujishima A., Hashimoto K., Watanabe T. (2000). Effects of the Surface Roughness on Sliding Angles of Water Droplets on Superhydrophobic Surfaces. Langmuir.

[94] Stanojevic L.P., Marjanovic-Balaban Z.R., Kalaba V.D., Stanojevic J.S., Cvetkovic D.J., Cakic M.D. (2017). Chemical Composition, Antioxidant and Antimicrobial Activity of Basil (*Ocimum basilicum* L.) Essential Oil. J. Essent. Oil Bear. Plants.

